# Potential Applications and Functional Roles of Exosomes in Cardiometabolic Disease

**DOI:** 10.3390/pharmaceutics13122056

**Published:** 2021-12-02

**Authors:** Sergio Ayala-Mar, Belén Rodríguez-Morales, Pedro Chacón-Ponce, José González-Valdez

**Affiliations:** Tecnologico de Monterrey, School of Engineering and Science, Av. Eugenio Garza Sada 2501 Sur, Monterrey 64849, Mexico; A00824101@itesm.mx (S.A.-M.); rdguezmorales.belen@gmail.com (B.R.-M.); A01566190@itesm.mx (P.C.-P.)

**Keywords:** exosomes, cardiovascular disease, metabolic disease, biomarker, drug delivery, biomaterials, therapeutic applications, regenerative medicine

## Abstract

Despite diagnostic and therapeutic advances, cardiometabolic disease remains the leading cause of death worldwide. Extracellular vesicles (EVs), which include exosomes and microvesicles, have gained particular interest because of their role in metabolic homeostasis and cardiovascular physiology. Indeed, EVs are recognized as critical mediators of intercellular communication in the cardiovascular system. Exosomes are naturally occurring nanocarriers that transfer biological information in the setting of metabolic abnormalities and cardiac dysfunction. The study of these EVs can increase our knowledge on the pathophysiological mechanisms of metabolic disorders and their cardiovascular complications. Because of their inherent properties and composition, exosomes have been proposed as diagnostic and prognostic biomarkers and therapeutics for specific targeting and drug delivery. Emerging fields of study explore the use exosomes as tools for gene therapy and as a cell-free alternative for regenerative medicine. Furthermore, innovative biomaterials can incorporate exosomes to enhance tissue regeneration and engineering. In this work, we summarize the most recent knowledge on the role of exosomes in cardiometabolic pathophysiology while highlighting their potential therapeutic applications.

## 1. Introduction

Cardiometabolic diseases are a cluster of disorders that include metabolic syndrome and its cardiovascular complications [1]. Worldwide, these diseases are the leading cause of death and a great economic and social burden [2]. Over the last decade, several therapeutics have been developed for treating metabolic disorders and preventing their cardiovascular complications [3]. Nevertheless, current therapeutic strategies are insufficient; thus, studying the regulation of cardiometabolic homeostasis and developing novel therapeutics remains of the upmost importance.

Extracellular vesicles (EVs) transfer bioactive proteins, lipids, DNA and RNA species between cells, contributing to the regulation of systemic homeostasis. Furthermore, because of the presence of EVs in almost all biological fluids and their remarkable functional role, EVs and exosomes, in particular, have been proposed as a diagnostic and prognostic biomarker, and as a disease mechanism in a diversity of fields that range from infectious diseases to neurodegenerative disorders [4,5].

In the cardiovascular system, evidence has shown that EVs contribute to the regulation of both physiological and pathophysiological processes [6]. Research into the role of EVs in metabolic and cardiovascular pathophysiology has revealed a complex intercellular communication network that can attenuate or sustain tissue injury [7,8]. In this context, EVs have been proposed as a disease screening tool, and as a novel therapeutic cardioprotection and tissue regeneration strategy [9].

Among EVs, exosomes are secreted by almost all mammalian cells in a size ranging from 30 to 150 nm [10,11]. These EVs are composed of a lipidic bilayer that contains transmembrane proteins such as integrins and tetraspanins (e.g., CD63, CD81 and CD82), and encloses soluble proteins, such as Rab, Annexin and components of the endosomal sorting complex required for transport (ESCRT), including ALIX, heat shock proteins (HSP) and TSG101 [12,13,14]. Furthermore, exosomes are enriched in DNA and RNA species that are remarkably stable and which can be later transferred to distant tissues [15,16].

Recent evidence shows that exosomes have a significant role in the metabolic syndrome pathogenesis and in its complications, mediating intercellular communication during the onset and progression of obesity, diabetes, hypertension and dyslipidemia, and the cardiac pathologies that often follow these disorders, such as atherosclerosis, acute coronary syndrome and heart failure [17].

Because of their functional role, composition and inherent properties, exosomes have been proposed as disease biomarkers that can also be used as bioactive molecule delivery vehicles [18,19]. Furthermore, exosomes can be isolated from specific sources or tailored to modulate a variety of pathological mechanisms [20]. For example, it has been shown that stem cell-derived exosomes could offer the possibility of cell-free regenerative medicine [21].

In this review, we update recent progress in exosome research focused on the functional role of exosomes in cardiometabolic pathology with a translational perspective. Here, we discuss exosome isolation and characterization advances and summarize the most significant roles of these EVs in the pathogenesis of the most common cardiovascular and metabolic disorders. Finally, this work highlights the potential applications of exosomes as biomarkers and for specific targeting and drug delivery, including insights into their use in innovative biomaterials and tissue regeneration.

## 2. Exosome Biogenesis, Isolation, and Characterization

Exosome research has become an expanding field that studies exosome biogenesis, isolation and characterization to elucidate exosome physiology and to find potential therapeutic applications. Evidence suggests that the unique features of these EVs can be directly attributed to their biogenesis. In contrast to other microvesicles, exosomes originate in the intracellular endosomal compartment [22]. To date, there appears to be a consensus that exosome biogenesis is dictated by the endosomal system.

In this process, early endosomes first form by the inward budding of the plasma membrane, then the endosomal membrane invaginates, generating intraluminal vesicles and forming mature endosomes or multivesicular bodies (MVBs) [23]. During this process, exosome cargo is sorted. Proteins in the plasma membrane and the cytoplasm are sorted into the exosome membrane and lumen, respectively [24]. DNA and RNA species are also sorted within the exosome lumen during endosome maturation [25].

The endosomal sorting complex required for transport (ESCRT) machinery, which consists of the ESCRT-0, -I, -II, -III subunits and associated protein complexes, such as syndecan-syntenin-ALIX and AAA ATPase Vps4, are critical for membrane remodeling, exosome cargo sorting and MVB intracellular transport [26]. In the ESCRT pathway, ubiquitinated cargoes are sorted into exosomes through phosphatidylinositol-3-phosphate (PI3P) and hepatocyte growth factor-regulated tyrosine kinase substrate (Hrs) interaction [27]. Then, tumor susceptibility gene 101 (TSG101) mediates ESCRT-I recruitment. ESCRT-I and ESCRT-II recognize specific endosomal domains that contain ubiquitinated proteins, promoting inward budding. ESCRT-III binds to ESCRT-II to cleave the bud of intraluminal vesicles using ATP catalyzed by Vps4 [28].

ALIX is an accessory ESCRT protein that is critical for exosome biogenesis. The ALIX-syndecan-syntenin axis mediates intraluminal vesicle formation and cargo sorting. For instance, ALIX facilitates the incorporation of tetraspanins into the exosomal membrane during late endosome formation [29]. Remarkably, ESCRT components, ALIX and TSG101 are also found in exosomes. However, evidence suggests that MVB biogenesis can also occur by an ESCRT-independent mechanism [23].

On their part, tetraspanins, which are transmembrane proteins enriched in the exosomal membrane such as CD9, CD63, CD81 and CD82, appear to mediate the biogenesis of intraluminal vesicles in the MVBs, and exosome cargo sorting release [30]. These proteins may form a platform, also known as the tetraspanin scaffold or web, that mediates the interaction of the bilayer membrane with other proteins promoting the budding process [31,32]. In addition, lipid molecules such as ceramide may also play essential roles in exosome biogenesis because of their intrinsic role in membrane remodeling [33]. Finally, sphingomyelinase, Rab GTPases, such as Rab27a, Rab2b, Rab5a and Rab91, and SNARE proteins play a crucial role in exosome secretion, mediating MVBs fusion with the plasma membrane and exosome release into the extracellular space [34].

Exosome transport and uptake within the body is not a random process and it appears to be dependent on transmembrane–protein interactions [35,36]. Upon reaching their target, membrane proteins in the exosomal membrane and in the plasma membrane of recipient cells can interact, inducing downstream signaling. Furthermore, these EVs can fuse with the plasma membrane of the target cells, which directly results in the release of their cargo into the cytoplasm or in exosome internalization by endocytic pathways [37].

The intracellular transport and subcellular fate of exosome cargo upon uptake remains to be fully elucidated. Various pathways have been proposed to dictate exosome fate after internalization, reflecting the multifaceted function of EVs. For instance, upon endocytosis, the endocytic pathway may mediate exosomal cargo transport to the trans-Golgi network and the endoplasmic reticulum [38].

Through the endocytic pathway, exosomal proteins can reach the plasma membrane via recycling endosomes or can be resorted into exosomes via late endosomes, allowing transfer of exosome cargo through distinct cell types until it reaches the target cell. Likewise, fusion of endocytosed exosomes with late endosomes may result in the release of exosome cargo directly into the cytoplasm of recipient cells, allowing bioactive molecules to reach their targets. For example, nucleic acids and proteins enclosed in exosomes are proposed to reach the nucleus via nucleoplasmic reticulum-associated late endosomes [39].

In this sense, labelling techniques have been crucial to better understand the uptake mechanisms and biodistribution of exosomes, enabling progress in the development of these EVs as an efficient delivery vehicle. Imaging methods for studying exosome biodistribution include bioluminescence and fluorescence labelling, radio isotope labeling and magnetic resonance [40]. However, because of their heterogenous composition and diverse origin, several factors influence exosome biodistribution, meaning that tailored imaging solutions are required for specific visualizations.

Exosome half-life in the blood stream is less than a few minutes, reflecting rapid tissue distribution or uptake by the mononuclear phagocytic system [41]. Circulating and tissue-resident phagocytic cells such as macrophages and neutrophils play critical roles in the clearance of exosomes. On the other hand, exosomes are retained for longer than 24 h in tissues that have high blood perfusion and well-developed phagocytic systems, such as the liver and the spleen [42].

The liver, spleen, kidney and the lungs are the major sites of exosome distribution when administered systemically. Indeed, the liver plays a prominent role in early and late clearance of exosomes. Evidence shows that renal excretion may not contribute to the rapid clearance of EVs from circulation [38]. However, biliary excretion and renal clearance of exosomes have been observed as soon as 5 h after administration [43].

One of the main factors that influence exosome biodistribution is their cellular origin. Exosomes from different cell types have shown an asymmetric biodistribution [42]. Furthermore, the biodistribution and retention of exosomes in a particular tissue can be influenced by pathophysiological processes [43]. In this regard, exosome surface modifications have allowed the attachment of different moieties for targeted drug delivery. Another approach has been to develop strategies to attenuate liver accumulation and clearance, such as exosome PEGylation [38].

**Figure 1:**
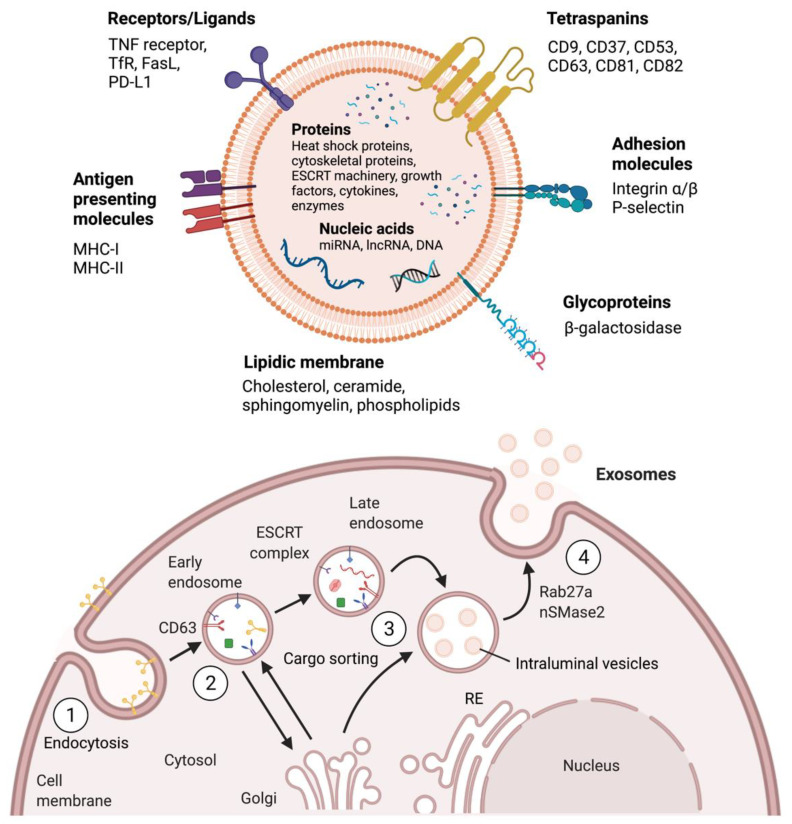
A simplified representation of exosome biogenesis and composition. (1) Exosome biogenesis begins by inward budding of the cell membrane. (2) Tetraspanins such as CD63 are proposed to mediate early endosome formation and transport. (3) The ESCRT complex and associated proteins sort proteins, DNA and RNA species into exosomes and are proposed to mediate late endosome formation. (4) Late endosomes are multivesicular bodies that fuse to the cell membrane and release exosomes to the extracellular space, a process that has been associated with Rab27a and nSMAse2 activity. Exosomes are formed by a lipidic bilayer with receptors, ligands, and transmembrane proteins, while soluble proteins, DNA and RNA species are enclosed in the lumen.

Exosome biogenesis, release and uptake are complex processes in which different pathways seem to converge. These processes appear to be highly conserved, which indicates the critical role of exosomes in cell physiology and tissue homeostasis. Figure 1 shows a simplified schematic representation of exosome biogenesis and composition according to the current consensus.

Regarding exosome isolation procedures, differential ultracentrifugation has been the most frequently used technique. It has been widely used to isolate exosomes from blood, saliva and cell culture medium, to mention some examples [44]. This approach employs a series of centrifugation steps at low speeds to remove cells, debris and larger EVs, followed by ultracentrifugation at ~100,000× *g* [45]. To further purify the sample, protocols that include a step of ultracentrifugation using an iodixanol or sucrose gradient have been developed [46,47]. Density-gradient centrifugation protocols appear to increase the purity of the exosome-enriched fraction by removing other EVs, whose size overlaps the size of exosomes [48]. However, total exosome yield can be compromised by additional ultracentrifugation steps, which are also time consuming.

In addition to differential ultracentrifugation, other approaches such as immunoaffinity capture, chromatographic separation, polymer-based precipitation, and microfluidic devices have been developed to obtain an exosome-enriched fraction. For instance, exosome isolation by immunoaffinity-employing beads coated with antibodies targeting either CD81, CD9 or CD63, all of them canonical exosomal markers, has been recently reported [49,50]. This method appears not to compromise exosome yield, while also increasing purity [51,52]. However, recent reports show variability in the expression of these exosomal markers; thus, antibodies must be carefully and selectively used.

Recovery of exosomes using polymers such as polyethylene glycol (PEG) facilitates exosome precipitation using low-speed centrifugation [53]. Precipitation protocols take advantage of volume-excluding polymers, which are mixed with the sample and incubated over short time periods, to generate an exosome-enriched fraction without the need of ultracentrifugation [54]. This method is considered to be rapid; however, it results in the coprecipitation of other EVs, lipids and molecular aggregates whose sizes and structures resemble that of exosomes [55].

**Figure 2:**
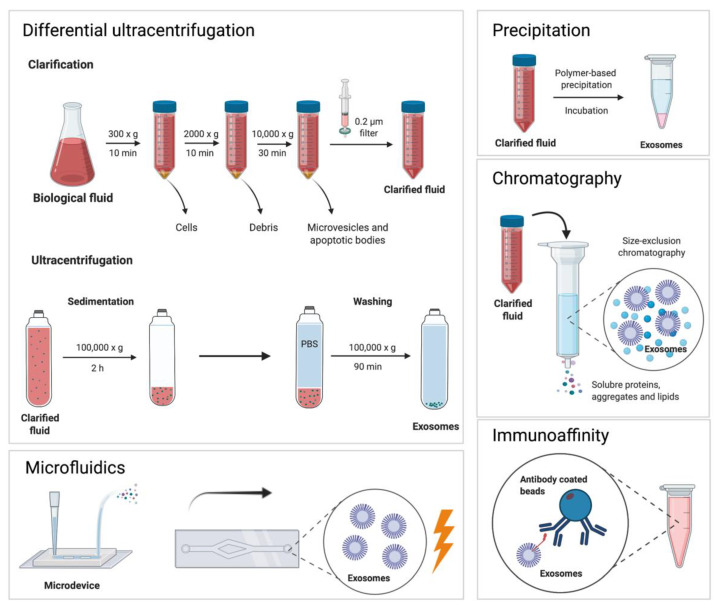
The most commonly used exosome isolation methods: Differential ultracentrifugation involves a series of centrifugation steps at low speed to remove larger particles. Afterwards, ultracentrifugation is used to pellet exosomes and to further purify the sample. Microfluidics, precipitation, chromatography and immunoaffinity approaches can provide a comparable yield and purity when compared to ultracentrifugation.

On their part, size exclusion chromatography, ion-exchange chromatography and immunoaffinity chromatography can also increase sample purity, without compromising exosome yield [56]. However, optimizing such protocols can be time consuming and requires specialized equipment, and reagents that are often built in-house. More recently, microfluidics have also gained attention for their exosome isolation capabilities. Microfluidic technology employs a variety of approaches that take advantage of the physicochemical properties of exosomes, as well as canonical exosomal markers, to increase isolation efficiency [57,58]. Microdevices have been used to simultaneously isolate and characterize exosomes [59]. However, developing such devices can be time consuming and laborious, meaning reproducibility is often compromised. Figure 2 depicts the most common exosome isolation techniques, including an overview of a general differential ultracentrifugation protocol.

The selection of the technique used to isolate exosomes is often directed by the intended downstream analysis and application. The physicochemical properties and molecular identity of the EV-enriched fractions are analyzed to validate exosome isolation and to determine purity and yield. These fractions are assessed for particle size, surface charge and morphology. Exosome and protein concentrations are also determined, while the expression of canonical exosomal markers is detected and quantified by proteomic analysis.

Exosome size can be determined by using dynamic light scattering (DLS) or nanoparticle tracking analysis (NTA) [60]. DLS can also be used to determine the particle surface charge or zeta potential. However, it may not be suitable for measuring complex biological samples with large size distributions where exosome concentration cannot be determined. On the other hand, NTA allows users to simultaneously determine both particle size and concentration with high resolutions [61]. The detection of exosomes by NTA technology is fast and exosomes can be observed in real time. However, contaminating proteins and detection thresholds can influence exosome quantification.

Furthermore, exosome concentration can also be determined by an enzyme-linked immunosorbent assay (ELISA) directed to canonical exosomal markers, correlating absorbance to particle size, employing standards analyzed by NTA [62]. The enzymatic activity of specific exosomal proteins can also be determined to infer particle number in the same aforementioned manner [63].

For its part, exosome morphology can be studied using electron microscopy, with transmission electron microscopy (TEM) being the most often reported technique [64]. Scanning electron microscopy (SEM) can also be used; however, TEM is preferred over SEM because the latter displays lower resolution on biological samples [65]. Cryogenic electron microscopy (Cryo-EM) has been reported to be superior to TEM or SEM to determine exosome morphology, size and concentration; however, because of its novelty and cost, this technique is scarcely reported [66].

Studying exosome morphology using electron microscopy reveals cup-shaped nanoparticles formed by a membrane that resembles the cell membrane. Electron microscopy can also be used to study particle size and concentration, although DLS and NTA are preferred for their higher sensibility and sensitivity. However, electron microscopy has revealed that the isolation technique influences exosome morphology, in addition to yield and purity [67].

A more general method to analyze exosome content is to measure the total amount of protein in the sample. For this purpose, the Bradford protein assay and directly measuring absorbance at 280 nm have been widely reported [68]. Nonetheless, this approach requires that the sample has not been contaminated by protein aggregates, which is common for most reported isolation techniques.

Finally, canonical exosomal markers are assessed by proteomic techniques to validate exosome isolation and to quantify their concentration. As mentioned, canonical exosomal markers include CD9, CD81 and CD63, TSG101, HSP70 and ALIX. For this, Western blot is the most often reported method to detect and quantify exosomal markers [14,66]. However, this is a semi-quantitative approach, and its use is usually limited to validating the presence of exosomes in the sample. On the other hand, flow cytometry has been used to detect and quantify exosomal markers, showing higher sensitivity and specificity than Western blot or ELISA [69]. However, exosomes are required to bind to beads functionalized with antibodies and thus require an additional step to further purify the sample after isolation and analysis. Furthermore, the optimization of flow cytometry protocols can be time consuming, mainly because of exosome size concerns.

It is important to consider that exosome research is a novel and expanding field and it is difficult to find consensus amidst methodological challenges and advances. Nonetheless, the achieved progress in exosome isolation and characterization has allowed the study of their function and potential applications, revealing a remarkable role in the pathogenesis of a variety of complex disorders such as cardiometabolic disease.

## 3. The Role of Exosomes in Metabolic Disease

Metabolic diseases include biochemical and physiological abnormalities in glucose and lipid metabolism, such as diabetes and dyslipidemia. These diseases are commonly associated with obesity and hypertension. Altogether, this disorder cluster encompasses the metabolic syndrome. The pathogenesis of this syndrome is a complex and multifactorial process that involves insulin resistance, adipose tissue redistribution and abnormal lipoprotein metabolism, amidst an inflammatory response [70]. It features hyperglycemia, hyperlipidemia, central adiposity and hypertension, and greatly increases cardiovascular risks [1].

Nonetheless, the clinical criteria for the diagnosis of metabolic syndrome have not yet been harmonized and its definition continues to be updated. Recent evidence shows that exosomes released by a myriad of cell types can modulate critical metabolic pathways and inflammation in the setting of metabolic disorders [70]. These EVs represent an opportunity to develop biomarkers for diagnosis and prognosis, as well as innovative therapeutic approaches for metabolic syndrome [71].

### 3.1. Obesity

Central adiposity is a well-known risk factor for the development of metabolic syndrome and its cardiovascular complications. Adipocyte hypertrophy has been associated with an increase in the level of adipocyte-derived exosomes in obese humans [72]. Furthermore, the microRNAs (miRNAs) enclosed in these exosomes differ between obese and lean individuals [73]. The exact mechanism whereby exosomes regulate obesity, and its complications, is not known. However, evidence suggests that exosomes contribute to glucose and lipid metabolism in the heart, skeletal muscle, liver and pancreas [74].

Recent evidence shows that EVs may link adiposity, insulin resistance and inflammation. For instance, adipocytes can regulate inflammatory pathways that contribute to a chronic inflammatory state and glucose intolerance. Deng et al. found that adipocyte-derived exosomes from obese mice stimulate macrophages to release IL-6 and TNF-α and induce their migration into adipose and liver tissue via the toll-like-receptor 4 (TLR-4) pathway [75]. In turn, adipose tissue macrophages can regulate systemic metabolism [76].

Ying et al. found that in obese mice, adipose tissue macrophages release exosomes enriched in miRNA-155 [77]. Exosomal miRNA-155 suppresses the adipogenic transcription factor peroxisome proliferator-activated receptor γ (PPARγ) and CCAAT/enhancer-binding protein β (CEBPβ), which partly mediate insulin sensitivity [77,78]. Remarkably, in vivo testing showed that adipocyte-derived exosomes from obese mice induce insulin resistance in lean mice [77].

Adipocyte-derived exosomes can regulate glucose and lipid metabolism in peripheral tissues, such as the liver, endothelial cells and skeletal muscle. These EVs induce the expression of tissue inhibitor of matrix metalloproteinase-1 (TIMP-1) and integrin αvβ-5, in hepatocytes and hepatic stellate cells, contributing to liver fibrosis [79]. Obesity is a known risk factor for nonalcoholic fatty liver disease, which may develop into nonalcoholic steatohepatitis (NASH), characterized by liver fibrosis.

The physiological context, mainly adiposity and nutritional status, seems to be a critical factor that determines the role of exosomes in metabolism. Adipocytes can release fatty acids to the systemic circulation via exosomes in addition to lipolysis [80]. Hepatic injury because of lipotoxic fatty acids results in the release of exosomes enriched in proinflammatory factors, that induce the expression of IL-1 and IL-6 in macrophages [75]. Likewise, during hyperglycemia, endothelial cell-derived exosomes carry miRNAs that accentuate the inflammatory response, increasing endothelial dysfunction which is a hallmark of metabolic disease [81].

Ectopic lipid accumulation in skeletal muscle is frequently observed during obesity, an excessive lipid uptake by myocytes increases exosome release [82]. These exosomes induce myocyte proliferation and differentiation and can be transferred to the pancreas and the liver. In the pancreas, exosomes from the skeletal muscle of obese individuals may influence ß-cell proliferation and function, contributing to insulin resistance [82]. These results suggest that adipose tissue regulates metabolic homeostasis partly through exosomes, and that these EVs may induce phenotypic variations in critical tissues, which in turn perpetuate metabolic deregulation and promote a systemic inflammatory state in obesity.

### 3.2. Diabetes Mellitus

It is known that adipocytes, skeletal muscle, hepatocytes and endothelial cells are sensitive to insulin. Activation of the insulin receptor increases glucose uptake, glycogen and protein synthesis. Insulin resistance results in free fatty acid mobilization, ectopic lipid accumulation, hyperglycemia and vasoconstriction [83]. Adipocytes, myocytes, hepatocytes and endothelial cells initiate and perpetuate these metabolic alterations, while triggering an inflammatory response.

Type-2 diabetes is characterized by hyperglycemia due to insulin resistance. High-glucose levels may induce the release of exosomes amidst glucotoxicity in a variety of cell types [84]. Recent evidence shows an increase in the level of circulating EVs from immune cells in type-2 diabetes patients. For instance, Botha et al. found a positive correlation in the level of monocyte-derived EVs and insulin resistance [85].

Exosomal miRNAs are proposed as important mediators of multiple metabolic functions. For instance, miRNAs such as let-7, miR-103, miR-29, miR-223 and miR-107 may be implicated in the pathogenesis of diabetes by mediating liver gluconeogenesis, insulin secretion and autophagy [86]. Indeed, exosomal cargo targets key proteins that are critical for insulin sensitivity and glucose metabolism.

Katayama et al. found that exosomal miR-20n-5p targeted AKT-interacting protein (AKTIP), which enhances AKT phosphorylation, reducing glycogen storage in skeletal muscle and promoting insulin resistance [87]. Exosomal miRNAs such as miR-155, miR-450b-3p and miR-151-3p also target signaling effectors of the insulin receptor, including proteins related to GLUT4 trafficking, FOXO1 and PPARγ [82].

In the context of hyperinsulinemia and hyperglycemia, adipocyte-derived exosomes induce a proinflammatory phenotype in macrophages, which in turn decrease the insulin receptor substrate 1 (IRS-1) and hormone-sensitive lipase (HSL) expression in adipose tissue [82]. In the liver, these proinflammatory macrophages target PPARγ expression, contributing to insulin resistance [82].

Several studies highlight the role of inflammation in the development of insulin resistance and type-2 diabetes [81,82]. In the pancreas, exosomal miR-142-3p and miR-142-5p promote a proinflammatory environment that may enhance β-cell apoptosis by triggering the expression of Ccl2, Ccl7 and Cxc100, which are involved in chemokine signaling and promote the recruitment of immune cells [88]. Likewise, a high palmitate diet stimulates myoblasts to release exosomes that are enriched in miR-16. This miRNA regulates the expression of the proliferation suppressor protein patched homologue 1 (PTCH1), which is implicated in pancreatic β-cell proliferation [89]. Furthermore, exosomal circular RNAs (circRNAs) have also been associated with diabetes progression by decreasing β-cell proliferation and insulin secretion [84].

### 3.3. Dyslipidemia

Dyslipidemia is characterized by high levels of triglycerides and total cholesterol, accompanied by a high concentration of low-density lipoprotein cholesterol and a low concentration of high-density lipoprotein cholesterol [90]. These abnormalities in lipoprotein metabolism correlate to the accumulation of lipid species that promote inflammation. Metabolically, active macrophages become activated by high levels of saturated free fatty-acids, insulin or glucose [91].

Exosomes partly sustain an overactive inflammatory response in metabolic disease [92]. During lipid overload, hepatocytes, skeletal muscle cells and adipocytes show an increased release of exosomes [73]. These EVs are enriched in ceramides, which can be transferred to myocytes and macrophages, promoting insulin resistance and inflammation [89]. Exosomes are also a source of bioactive eicosanoids critical for the inflammatory response [93]. In the liver, EVs released during hyperlipidemia have shown to induce inflammatory fibrotic phenotypes in hepatocytes, which have been associated with non-alcoholic steatohepatitis [73].

**Figure 3:**
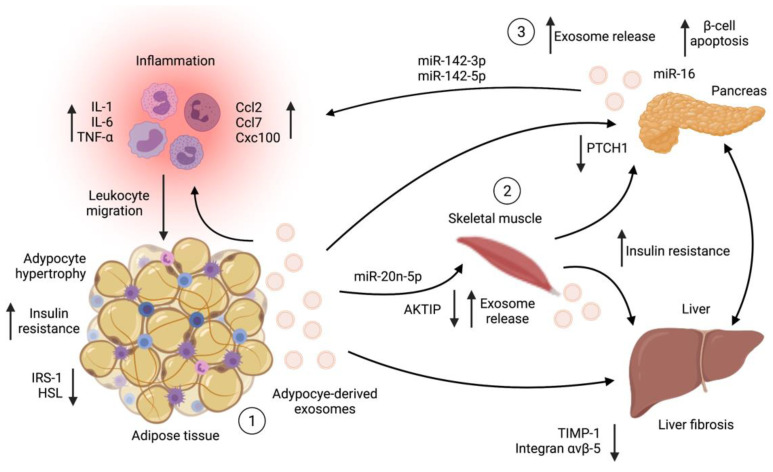
Exosomes as mediators of systemic metabolism in obesity and diabetes. (1) Adipose tissue can regulate systemic metabolism. In obesity, adipocyte-derived exosomes are released in response to adipocyte hypertrophy. (2) These EVs can induce insulin resistance in skeletal muscle, liver, and pancreas, inhibiting key regulatory signaling pathways that induce exosome release, collagen deposition and apoptosis. (3) In turn, these tissues perpetuate metabolic alterations, establishing a network of intercellular communication in which exosomes can transfer RNA species that promote inflammation and metabolic stress.

Recent evidence suggests that exosomes may influence lipid degradation and adipose tissue redistribution. For instance, circulating exosomes have been shown to also modulate the expression of lipid transporters, such as reverse cholesterol transport mediated by ABCA1 [94]. Furthermore, systemic metabolic abnormalities are seen during obesity, diabetes and dyslipidemia. These reflect the interplay between critical metabolic tissues that takes place in the pathogenesis of the metabolic syndrome. Exosomes from a variety of cells partly mediate intercellular communication in the context of obesity and its related metabolic alteration, as seen in Figure 3.

## 4. The Role of Exosomes in Cardiovascular Disease

Cardiovascular disease is a frequent complication of metabolic disorders. Exosomes from cardiomyocytes, cardiac fibroblasts, endothelial cells, cardiac-derived progenitor cells and cardiosphere-derived cells play a fundamental role in the maintenance of physiological homeostasis in the cardiovascular system [95]. Homotypical and heterotypical exosome transfer appears to be fundamental for communication in a multicellular system, such as the heart [96]. Recent evidence suggests that a variety of cell types in the cardiovascular system employ exosomes to modulate heart function during the onset and progression of cardiovascular disease [97]. In this section we will present recent evidence on the role of exosomes in the pathogenesis of vascular disease and heart dysfunction.

### 4.1. Atherosclerosis

Atherosclerosis is the leading cause of coronary artery disease and myocardial ischemic injury [98]. EVs have been found in atherosclerotic plaques at all stages of the disease [99]. Furthermore, the source of EVs in atherosclerotic lesions appears to be multicellular. Intensive tissue remodeling takes place in an inflammatory microenvironment during the onset and progression of this disease [100]. Plaque rupture or erosion with subsequent thrombosis is the ultimate consequence of atherosclerosis, which leads to stable or unstable ischemic heart disease and acute coronary syndrome [101].

Evidence shows that exosomes exhibit both anti-inflammatory and proinflammatory activity dependent on tissue homeostasis. Studies on the role of circulating exosomes derived from atherosclerotic lesions on inflammatory pathways in vivo are limited. However, in vitro observations have shown that exosomes increase local and systemic inflammation by promoting the release of inflammatory cytokines from immune and endothelial cells at early stages of plaque formation [102]. Exosomes also promote endothelial dysfunction by decreasing the release of nitric oxide from endothelial cells, inducing alterations in vascular tone and limiting the ability of the endothelium to limit plaque formation [103]. Furthermore, in vitro evidence has shown that EVs partly mediate lesion progression, calcification and rupture [104].

### 4.2. Ischemic Heart Disease and Acute Coronary Syndrome

Cardiomyocyte-derived exosomes are enriched in miR-133a and miR-1 during the onset of ischemic myocardial injury [105,106]. These miRNAs have shown cardioprotective effects by limiting cardiomyocyte hypertrophy. Elevated levels of these exosomes have also been found in the systemic circulation upon myocardial injury; thus, cardiomyocytes may transfer biological information to critical organs, such as the kidney or the bone marrow, as a protective mechanism against ischemic injury [107].

Evidence from multiple models of cardiac disease has shown that upon ischemia, cardiomyocyte-derived exosomes are enriched in other cardioprotective miRNAs such as miR-214, which prevents apoptosis and loss of contractility, and miR-30a, which regulates autophagy [108,109]. Thus, acute cardiac stress induces the release of cardiomyocyte-derived exosomes with a protective role, as a mechanism to attenuate the deleterious effects of hypoxia and oxidative stress. On the other hand, chronic cardiovascular stress, as in heart failure, atherosclerotic disease, and cardiomyopathy, stimulates cardiomyocytes to release exosomes that further propagate tissue damage by sustaining inflammation and promoting fibrosis.

Nonetheless, circulating EVs during acute coronary syndrome also have deleterious effects. Immune cells, erythrocytes, platelets and endothelial cells have been shown to release exosomes that promote endothelial dysfunction and oxidative stress in this setting [110,111]. Recent evidence shows that danger-associated molecular patterns in exosomes derived from immune cells may be associated to the complications of ischemic myocardial injury [112].

Ischemia-reperfusion injury is a hallmark of myocardial infarction, and it is a potent factor that increases exosome release in bone marrow-derived dendritic cells and macrophages [112]. In an inflammatory environment, dendritic cells downregulate the immune response by increasing the differentiation of CD4+ T cells into a Foxp3+ Treg anti-inflammatory phenotype via exosome signaling [113,114]. Exosomal HP70 also released by dendritic cells stimulates the PI3K/mTOR axis regulating Treg and Th17 cell differentiation and attenuating ischemia-reperfusion injury [113].

Conversely, endothelial cells release miR-155 in exosomes, which induces macrophage polarization into an inflammatory phenotype [115,116]. Macrophage-derived exosomes transfer miR-155 to cardiac fibroblasts, increasing inflammation and reducing cardiac function [117]. Altogether, these findings suggest that during acute coronary syndrome, rapid adaptive responses from cardiomyocytes aim to reduce cardiac dysfunction, while cardiac fibroblasts, immune cells and endothelial cells regulate the inflammatory response, either resolving or perpetuating inflammation.

### 4.3. Pulmonary Hypertension and Hypertensive Disease

Pulmonary arterial hypertension (PAH) is the result of pulmonary vascular remodeling characterized by vascular smooth muscle cell and endothelial cell increased proliferation [118]. High resistance and low flow in pulmonary vasculature result from vascular stiffening and thickening. Endothelial and vascular smooth muscle cell dysfunction are the hallmark of PAH. Interestingly, intercellular communication between vascular smooth muscle cells and endothelial cells might dictate the pathological features of this disease. [119]. Exosomes derived from vascular smooth muscle cells have been found to be enriched in miR-143, which induces endothelial cell proliferation and migration, increasing microvessel density [120]. Furthermore, PAH increases the release of exosomes from endothelial cells.

Hypertension is a clearly defined risk factor for cardiovascular diseases, and it is characterized by vascular remodeling and increased arterial resistance [121]. Progression of this disorder is mediated by inflammation and oxidate stress in the setting of the activation of the renin-angiotensin-aldosterone system (RAAS) [122]. Hypertensive disease is characterized by cardiac pressure overload. In this context, cardiomyocyte-derived exosomes are enriched in the angiotensin II type I receptor, which is transferred to other cardiomyocytes and vascular smooth muscle cells, increasing their adaptability to changes in blood pressure [123].

Conversely, evidence shows that macrophage-derived exosomes transfer MAPK and ICAM-1 to endothelial cells, which induce endothelial dysfunction in the setting of cardiac pressure overload [124]. It should be noted that exosome research in this field is incipient and further evidence is required to assert the role of exosome signaling on hypertensive disease. Nonetheless, exosome cargo appears to be enriched with proteins and miRNAs that regulate the RAAS system and that may induce important adaptations to changes in vascular tone [125].

### 4.4. Heart Failure

The hallmark of heart failure is cardiac remodeling as a consequence of myocardial infarction, coronary artery diseases and high blood pressure, which ultimately lead to heart dysfunction [126,127]. Cardiac remodeling ultimately results in cardiomyocyte hypertrophy and myocardial fibrosis [128]. Cardiomyocyte enlargement increases inflammation, and cardiomyocyte-derived exosomes are enriched in proinflammatory and apoptotic factors in this setting [129]. Cardiac hypertrophy can also be induced by cardiac fibroblast-derived exosomes upon cardiac dysfunction [130].

During cardiac remodeling, cardiac fibroblasts transfer exosomal miR-21-3p to cardiomyocytes, downregulating SORBS2 and PDLIM5 and leading to cellular hypertrophy [131]. Likewise, Spp1 and EGFR enclosed in cardiac fibroblast-derived exosomes upregulate the PI3K/AKT and MAPK signaling axis in cardiomyocytes [132]. Cardiac fibrosis can also be driven by cardiomyocyte-derived exosomes carrying HSP90, which plays a pivotal role in the activation of JAK/STAT signaling in cardiac fibroblasts [133]. Indeed, exosomal HSP90 and IL-6 induce the production and deposition of collagen in cardiac tissue by cardiac fibroblasts upon inflammation [134].

These findings suggests that exosomes mediate interactions between cardiomyocytes and cardiac fibroblasts that accentuate cardiac hypertrophy, inflammation, and fibrosis in the context of heart failure. Furthermore, homotypical and heterotypical exosome transfer partly mediates a feedback loop that induces tissue damage in repose to various forms of stress in the heart. Conversely, physical activity upregulates the level of exosomal miR-29b and miR-455 derived from cardiomyocytes, which downregulate MMP9 and reduce fibrosis in cardiac tissue [135,136].

**Figure 4:**
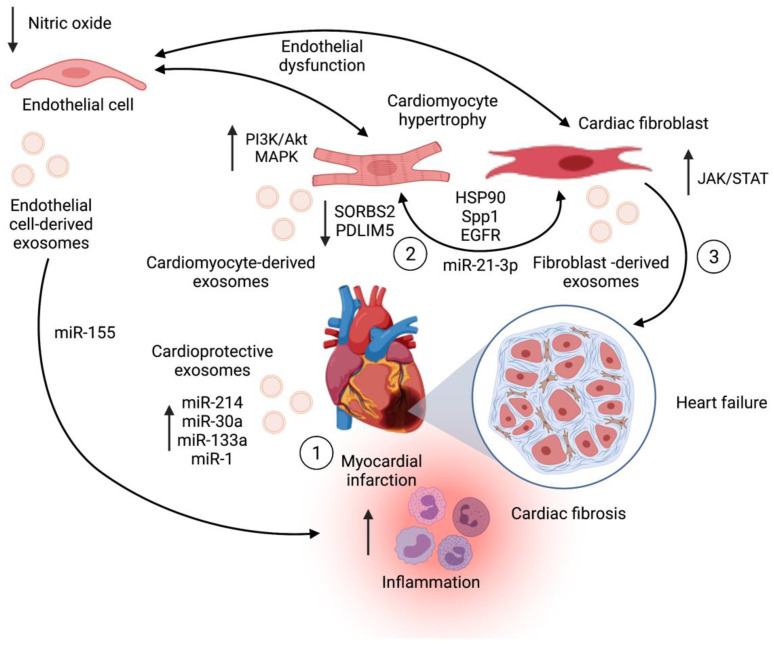
Cardiomyocyte-derived exosomes can either ameliorate or worsen myocardial injury during ischemia. (1) In the onset of myocardial infarction, cardiomyocyte-derived exosomes release exosomes enriched in miRNAs with a cardioprotective function. However, during heart failure, these cells release exosomes that increase cardiac hypertrophy and fibrosis. (2) In this setting, cardiomyocyte-derived and cardiac fibroblast-derived exosomes transport proteins and miRNAs that target critical signaling pathways in cardiomyocyte hypertrophy and fibroblast activation. (3) Cardiomyocyte-derived and cardiac fibroblast-derived exosomes contribute to collagen deposition and cardiac remodeling in heart failure. Endothelial dysfunction is mediated partly by endothelial cell-derived exosomes that promote inflammation and cardiac dysfunction in this context.

Endothelial dysfunction is also a hallmark of heart failure [126,128]. Endothelial cells also release exosomes that reflect cellular stress. For instance, proinflammatory cytokines such as TNF-α stimulate endothelial cells to release exosomes enriched in molecules related to superoxide protection and that modulate the nuclear factor κB pathway [137,138]. Exosomes can also regulate angiogenesis in the context of cardiac pathologies. However, the role of endothelial cells-derived exosomes on heart failure remains to be fully elucidated. As seen in Figure 4, exosomes mediate a dynamic regulation of cardiac function during myocardial infarction and heart failure, in which a variety of cells may release exosomes either with a cardioprotective function or as part of a mechanism that further increases tissue injury.

### 4.5. Cardiomyopathy

Cardiomyopathies are a group of diseases that impair the myocardium, ultimately leading to cardiac dysfunction and failure [139]. Inflammation is also a hallmark of cardiomyopathy and exosomes appear to play a proinflammatory role in this pathology. In the context of systemic inflammation, platelet-derived exosomes are enriched in NADPH oxidase, disulfide isomerase and nitric oxide synthase, impairing the resolution of the inflammatory process in the heart [140]. These exosomes also downregulate miR-223, which is an anti-inflammatory in cardiac tissue [140]. Endothelial cells may be a critical target of platelet-derived exosomes in this setting, since these EVs also induce apoptosis of the endothelium leading to vascular dysfunction [141].

Upon hyperglycemia, cardiomyocyte-derived exosomes are enriched in miR-320 which inhibits endothelial cell proliferation and perpetuates cell dysfunction in the onset of diabetes-associated cardiomyopathy [142]. Cardiac remodeling and fibrosis can also be associated to exosomal non-coding RNAs in cardiomyopathy. For instance, miRNA-1 and miR-133a are also enriched in cardiomyocyte-derived exosomes in this setting [143,144].

### 4.6. Valve Disease

Valve disease is a multifactorial process that can lead to cardiac dysfunction upon progression [145]. It can appear at any age because of a variety of mechanisms that include cardiac and systemic disorders. For instance, calcific valve disease is the most common cause of valve dysfunction, and it appears as a consequence of aging [146]. The role of exosome signaling in valve disease has not been fully studied. However, the levels of extracellular vesicles have been proposed as a biomarker for valve disorders such as calcific aortic valve disease and myxomatous mitral valve disease, and as a prognostic biomarker after surgical aortic valve replacement [147].

### 4.7. Disorders of the Rhythm

The role of exosomes in arrhythmia may be related to miRNAs that regulate calcium signaling and, thus, cardiac conduction and contraction. For instance, cardiomyocyte-derived exosomes are enriched in miR-1, miR-133 and miR-328 after ischemia, a known proarrhythmic factor [148,149,150]. These miRNAs regulate the function of key regulatory proteins of calcium signaling, such as Ca^2+^/calmodulin-dependent protein kinase II and L-type calcium channels, which regulate cardiac conduction and action potentials [151]. Furthermore, platelet-derived exosomes also present increased levels of these miRNAs, suggesting that exosomes from multiple cell types may impair cardiac conduction [152].

## 5. Potential Applications of Exosomes in Cardiometabolic Diseases

**Figure 5:**
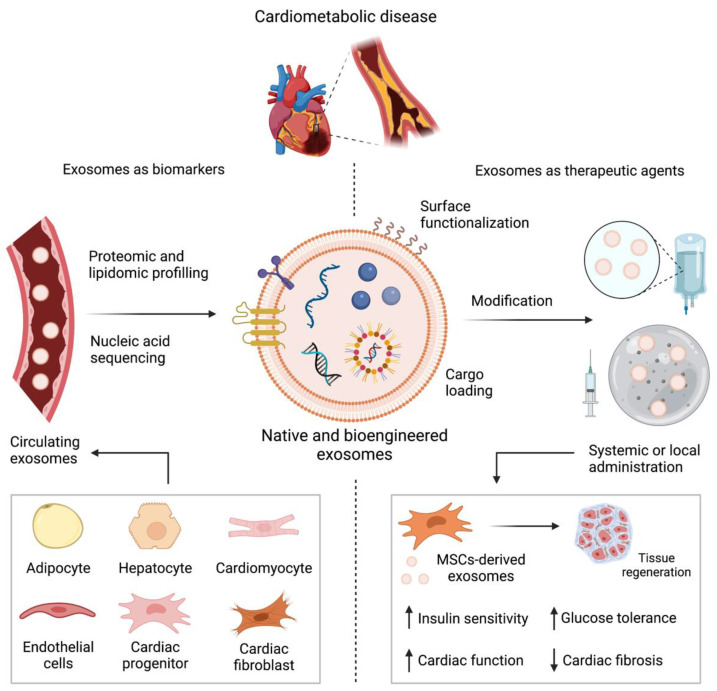
A schematic representation of the therapeutic applications of exosomes in cardiovascular medicine. In the context of cardiovascular and metabolic pathophysiology, circulating exosomes derived from a variety of cells can be used for biomarker screening. Native or synthetic exosomes can be modified either by surface functionalization or cargo loading, amongst other strategies. These bioengineered exosomes can be administered by systemic infusion or local injection using innovative biomaterials. Exosomes as therapeutic agents have shown promising results restoring critical parameter of cardiometabolic homeostasis. For instance, mesenchymal-stem cell derived exosomes have been proposed for tissue regeneration strategies.

Recent progress in our understanding of the pathogenesis of cardiometabolic maladies has led to extensive research on the use of exosomes as diagnostic and prognostic biomarkers, nanocarriers for specific drug delivery and cell-free therapeutics for tissue regeneration, as seen in Figure 5. In this context, Huda et al., found 205 clinical trials related to exosome research up until January 2021, 100 of which included cardiometabolic diseases or their complications [153].

Exosomes are highly biostable and reflect the state of tissue homeostasis. Furthermore, because of their intrinsic properties, exosomes have been proposed as biomarkers that can be obtained by noninvasive methods and that can be used for the early detection of disease and to assess the efficacy of therapeutic interventions [154]. Indeed, exosomes are proposed as drug nanocarriers because of their biocompatibility, biodegradability, specificity, and stability. Exosomes can also be loaded with therapeutic compounds and have shown selective delivery to target tissues or cells [155,156].

As it can be seen, EVs are naturally occurring nanocarriers. However, exosomes can be modified, or synthetic exosomes can be engineered to improve cargo loading and delivery efficacy. Incorporating bioactive molecules into exosomes is the most widely reported exosome modification for specific delivery [157]. Furthermore, parental cells can be modified by gene editing so that exosomes released from these cells already enclose bioactive molecules. Furthermore, different therapeutics can also be loaded into exosomes after isolation [153]. On the other hand, exosome surface modifications that can either change the surface charge or molecular composition are also being explored [158].

Tissue engineering and cell therapy for tissue regeneration are developing fields that have encountered a variety of complications [159]. Over the years, the efficiency of these therapeutic approaches for tissue regeneration has been relatively low. Because of their properties, exosomes may potentially improve the efficacy and safety of these therapeutic strategies. For instance, exosomes derived from mesenchymal stem cells (MSCs) have shown remarkable efficacy for the regeneration of a variety of tissues [160]. Likewise, exosomes can be incorporated into innovative biomaterials to develop cell-free delivery platforms for tissue engineering.

### 5.1. Exosomes as Biomarkers

#### 5.1.1. Metabolic Diseases

RNA sequencing, in particular the identification of exosomal miRNAs, piwi interacting RNAs (piRNAs) and long non-coding RNAs (lncRNAs), is a potential biomarker for the diagnosis and prognosis of cardiometabolic disease. Evidence shows that measuring the differential expression of exosomal and total plasma RNA species separately can provide a more specific platform for identifying novel biomarkers. [161,162].

Exosomal miRNAs may be useful in identifying the individual risk of developing diabetes in obese patients. The level of expression of exosomal miR-23a-5p, miR-6087 and miR-6751-3p has shown to be an indicator of diabetes in obese patients, while exosomal let-7b, miR-146a, miR29c, miR-222/223 and miR-26b have been associated with body mass index, dyslipidemia and values of insulin resistance [163,164]. Furthermore, exosomal miRNAs have the potential to improve insulin resistance by modulating the adiponectin pathway. In fact, exosomal miR-326, let-7a and let-7f have been found to regulate this pathway in diabetic patients [164]. Thus, these miRNAs are potential therapeutic targets that may have anti-atherogenic and anti-inflammatory effects.

Cardiovascular complications in patients with metabolic syndrome or diabetes are common. Microvascular damage from metabolic stress ultimately results in decreased tissue perfusion. Circulating exosomal miR-378g, miR-556-3p, miR-151a-3p, miR-130b-5p, miR-4488 and miR-183-5p have been related to early vascular complications of metabolic disorders [163]. These may serve to indicate the onset of endothelium damage and can be proposed as biomarkers for prognosis and to assess therapy efficacy. Likewise, urine-derived exosomes can be proposed as biomarkers of early-stage kidney disease.

The level of exosomal miR-26a-5p, miR-191-5p, miR-222-3p, miR-126-3p in urine has been associated to albuminuria and can be proposed to assess kidney function and nephropathy progression in diabetic and hypertensive patients [165]. Delic et al., found that 14 exosomal miRNAs show differential expression in healthy patients and patients with diabetes with or without diabetic nephropathy [166]. In particular, miR320c can be an indicator of a decreased glomerular filtration rate because of its regulatory role in the transforming growth β-signaling pathway. Exosomes can also be a tool to assess the efficacy of therapeutic interventions such as bariatric surgery, which improves cardiometabolic health by restoring adipose tissue homeostasis. In fact, Witczak et al., showed that exosomes could partly mediate this process as the level of circulating EV-derived fatty acid binding protein 4 (FABP4) exhibits a transient increase after surgery [167].

Obesity has been found to change the size and miRNA content of adipocyte-derived stem cell (ASC)-derived EVs. Differential expression analysis of these EVs allows us to distinguish between lean and obese patients, identifying upregulated miRNAs that mediate the inflammatory response and downregulated miRNAs implicated in cell proliferation and angiogenesis, in obese patients. [168] Castaño et al. found that obesity changes the miRNA profile of plasma exosomes in mice, including increases in miR-122, miR-192, miR-27a-3p and miR-27b-3p [73]. Likewise, exosomal miR-20b-5p has been associated with type-2 diabetes in humans [88]. The level of exosomal proteins such as CD9, CD62 and TSG101 that are canonical exosomal markers has been associated with diabetes-associated calcification and aging [169]. Indeed, circulating exosomal miRNAs may potentially be used as biomarkers and therapeutic targets in patients with obesity and with or without diabetes [163].

#### 5.1.2. Cardiovascular Diseases

RNA species found in total plasma can be an indicator of tissue damage but exosomal specific RNAs have been more easily associated to specific pathologies and their progression. Circulating exosomal miRNAs such as miR-146a and exosomal piRNAs such as has-pIR-0200009 and has-piR-006425 are differentially expressed in healthy volunteers when compared to heart failure patients and have been proposed as biomarkers for the diagnosis of cardiac dysfunction due to a reduced ejection fraction [161,170]. Furthermore, circulating exosomal miR-92b-5p may be used to identify reduced ejection fraction patients with acute heart failure, while exosomal miR-425 and miR-744 may predict cardiac fibrosis [171,172].

The accurate diagnosis and prognosis of acute coronary syndrome greatly influence clinical decisions and outcomes. Exosomal miR-126, miR-21 and miR-204 are more highly expressed in patients with unstable angina and acute myocardial infarction when compared to healthy patients. Furthermore, the circulating level of miR-126 has been found to be positively correlated with the severity of coronary artery stenosis [173]. Lower levels of miR-340 and miR-424 and higher levels of miR-340 have also been found in patients with ischemic stroke and myocardial infarction [174]. These exosomal RNA species are potential biomarkers for the diagnosis of acute coronary syndrome or ischemic cardiovascular events in patients without established cardiac disease, while measuring the circulating level of exosomal miR-1915-3p, miR-4507 and miR-3656 may be useful to diagnose acute myocardial infarction in patients with stable coronary artery disease [175]. Regarding long-term cardiovascular risk assessment, promising results suggest that elevated plasma levels of exosomal miR-133a of patients with familiar hypercholesterolemia predicts cardiovascular events within the next 2 years [176].

Screening circulating long non-coding RNAs (lncRNAs) in acute myocardial patients has revealed that these RNA species also relate with clinical parameters, inflammatory biomarkers, prognostic indicators and myocardial damage [177]. For instance, Wang et al. found that exosomal lncRNA HIF1A-AS1 could act as potential biomarkers for atherosclerosis [178]. The protein content of circulating exosomes also reveals distinct protein signatures in the pathogenesis of a variety of cardiovascular diseases. For instance, the level of expression of exosomal NEAT1, MMP-9 and PTEN has been found to be elevated during acute myocardial infarction [173,179]. On their part, Otero-Ortega et al. conducted comparative proteomic analysis of EVs between patients with ischemic stroke, myocardial infarction and healthy controls and found differences in the expression profile of apolipoprotein B, alpha-2 macroglobulin and fibronectin [174].

A pioneering study by Moreno et al., found that the composition of the microbiome in patients with coronary artery disease may dictate clinical outcomes after cardiovascular events [180]. These microbial communities may release EVs during the onset of myocardial injury, which, depending on their origin, could be enriched in MMP9 and elicit a protective role during plaque rupture [180]. Likewise, Ni et al. characterized the protein profile of serum exosomes from atrial fibrillation patients and found 40 elevated proteins and 75 reduced proteins when compared to healthy controls [181]. Interestingly, these proteins were associated with anticoagulation, the complement system and protein folding.

RNA sequencing and proteomic analysis of urinary exosomes has revealed an exosome signature in the end-organ damage seen during hypertensive disease [182]. For instance, miR-26a, miR-146a and miR-596 have been associated with early kidney damage, albuminuria and a poor prognosis [182,183]. Indeed, over 48 exosomal proteins and 27 exosomal miRNAs have been found to be differentially expressed between hypertensive patients and healthy controls [184,185]. These are potential targets that could facilitate the diagnosis, prevention and treatment of hypertensive disease.

### 5.2. Drug Delivery Systems for Specific Targeting towards Cardiometabolic Diseases

Exosomes are recognized as biomaterials that can be used for the control and sustained delivery of bioactive compounds. As has been mentioned, depending on their cellular source and the homeostatic state of their origin tissue, evidence has shown that exosomes may enclose proteins and RNA species that show protective properties for cardiometabolic diseases and their complications.

For instance, it has been shown that exosomal miR-690 derived from M2 polarized bone marrow-derived macrophages improves insulin sensitivity and glucose tolerance in an obese mice model [186]. Myocyte-derived exosomal miRNAs from trained mice have been shown to improve insulin sensitivity and improve glucose tolerance in sedentary mice [187]. Furthermore, skeletal muscle cell-derived exosomes transfer miR-130a to endothelial cells, regulating capillary refraction caused by type 2 diabetes in vitro [188].

MSC-derived exosomes also carry miRNAs that have been identified as potential therapeutics for metabolic diseases [143,144]. Exosomes carrying miR-21, derived from MSCs, can protect beta cells against apoptosis induced by hypoxia, reducing endoplasmic reticulum stress and inhibiting p32 mitogen-activated protein kinase [189]. MSC-derived exosomes have also shown to reduce hyperglycemia by increasing glycolysis and inhibiting gluconeogenesis in type 2 diabetes mellitus models in vivo and in vitro [190]. Also, ASC-derived exosomes may regulate inflammation in white adipose tissue [191]. These EVs can also be used to treat complications associated to diabetes and obesity. For instance, miR-486 enclosed in these EVs has been found to alleviate diabetic nephropathy in vivo, while bone marrow MSC-derived exosomes improve bone regeneration, which is impaired in obesity [149,150].

Circulating exosomes and exosomes derived from adipose tissue may also be enriched with miRNAs that regulate metabolic function in obesity and diabetes. In particular, it has been found that exosomes derived from healthy individuals and brown adipose tissue reduce body weight, lower blood glucose and alleviate lipid accumulation in vivo [150,151,152]. Nonetheless, modifications to exosome content and structure can generate a more efficient delivery system, which can be tailored to target specific cell types in the appropriate clinical setting.

Melatonin-pretreated MSC-derived exosomes promote diabetic wound healing by targeting the PTEN/AKT pathway [192]. Likewise, atorvastatin treatment induces the release of exosomes from MSCs that promote diabetic wound repair [193]. Exosomes derived from mmu_circ_0000250-modified adipose-derived MSCs promote wound healing in diabetic mice by inducing miR-128-3p/SIRT1-mediated autophagy [194]. On the other hand, knock-outs of miR-155 in adipose tissue macrophages result in exosomes that improve glucose tolerance and insulin sensitivity in obese mice [78]. Furthermore, inhibition of miR-15a-3p from diabetic patients’ blood-derived exosomes through knockdown accelerates diabetic wound repair in vivo and in vitro by activation of the NOX5/ROS signaling pathway [195].

Mentkowski et al. engineered cardiosphere-derived cells that release exosomes containing Lamp2b, a transmembrane exosomal protein, fused to a cardiomyocyte specific peptide [196]. These exosomes resulted in an increased exosome uptake and decreased cardiomyocyte apoptosis and higher cardiac retention. Likewise, Li et al. encapsulated Ldlr mRNA into exosomes by forced expression of Ldlr in AML12 cells; these exosomes significantly lowered the level of low-density lipoprotein cholesterol and reduced lipid deposition in the liver in a mice model of familiar hypercholesterolemia [197].

Furthermore, gene-therapy strategies might be improved by targeted delivery using exosomes. These vesicles can be engineered to target a specific subpopulation of cells within a tissue, while also containing specific bioactive molecules directed to critical pathways in the pathogenesis of cardiometabolic disease. For example, synthetic exosomes have been generated by constructing an HDAd that expresses an antagomiR directed at miR-33a-5p and that also enhances uptake of anti-miR-33a-5p into exosomes [198]. These exosomes reduced miR-33a-5p expression and increased apoAI-mediated cholesterol efflux in intimal and medial cells. Likewise, exosomes carrying miRNA-181a-overexpressing lentiviruses have improved cardiac function after myocardial ischemia-reperfusion injury in a mouse model of myocardial infarction [199]. In another example, to increase exosome uptake and cardiac retention, Liu et al. developed magnetic nanoparticles conjugated to antibodies against CD63 and myosin-light-chain surface markers. A local magnetic field guided the accumulation of these nanoparticles in infarcted cardiac tissue, leading to a reduction in infarct size and improving cardiac function after myocardial infarction [200].

### 5.3. Exosomes for Regenerative Medicine

Over the last decade, mesenchymal and stromal stem cells have shown promising results for tissue regeneration in cardiometabolic disease. Stem cell therapy has shown cardiac regeneration and improved outcomes of ventricular function in animal models of cardiovascular disease. This approach has shown promising results, ameliorating ischemia-reperfusion injuries and heart failure after myocardial infarction. However, stem cell therapy has faced important challenges, such as cell differentiation, viability and precise delivery to target tissues.

Evidence suggests that MSCs exert their effects through exosomes [201]. Stem-cell derived exosomes can be proposed as an effective companion of cell therapy, since these EVs prevent immune rejection after stem cell implantation by modulating the immune response and increasing the cardiac retention of therapeutics [202,203,204]. Furthermore, exosomes represent a cell-free alternative for tissue regeneration and engineering.

In animal models, MSC-derived exosomes have reduced infarct size and inflammation, inhibiting the effects of myocardial ischemia-reperfusion injury. [202,203,205]. Evidence shows that MSC-derived exosomes hold the potential to modulate the immune response, ameliorating the deleterious effects of an overactive immune system during ischemia-reperfusion injury [206]. This potential has been found to be mediated by miRNAs such as miR-193a-5p, mmu-miR-7116-5p, miR-146a-45p, miR-182 and miR-24 [204,207,208,209,210].

It has also been shown that a higher concentration of circulating exosomes protects endothelial cells from oxidative stress damage [209]. Genetic engineering can be used to upregulate or downregulate the expression of specific proteins and RNAs species in stem cells, which release modified exosomes accordingly. For instance, a knock-out of beta-2 microglobulin in MSCs enhances exosome-mediated cardiac regeneration and inhibits cardiac fibrosis, improving cardiac function after myocardial infarction [204]. Likewise, CXCR4-overexpressing cardiac-resident progenitor cells and SDF1 overexpressing MSCs have been generated to release exosomes that protect cardiac function and inhibit myocardial tissue damage after infarction [206,211].

Another approach is to modify MSC-derived exosomes with monocyte mimics through membrane fusion. These modified exosomes have exhibited enhanced targeted efficiency by mimicking monocyte recruitment after cardiac injury, improving therapeutic outcomes in cardiac function [212]. Indeed, exosomes have been delivered into cardiac tissue either via intravenous infusion, intramyocardial injection or intrapericardial injection [203,206,207,208].

Allograft transplantation of myogenic progenitor cell-derived exosomes have restored critical heart protein expression and has improved cardiac function in a Duchenne Muscular Dystrophic mice model [203]. Moreover, delivery systems for the local delivery of therapeutics, such as hydrogel and scaffold systems, have been employed to increase exosome uptake and cardiac retention.

Furthermore, MSC-derived exosomes encapsulated in alginate and functional peptide hydrogels have significantly improved cardiac function and reduced inflammation, fibrosis and apoptosis after myocardial infarction [213,214,215]. In this context, Yao et al. have recently developed a minimally invasive spray based on MSC-derived exosomes and biomaterials that improves cardiac function, reduces fibrosis and promotes angiogenesis after myocardial injury [215]. Human umbilical cord-derived MSC-derived exosomes and adipose-derived SC-derived exosomes have also been loaded into hydrogel systems and have been shown to promote diabetic wound repair [177,178,179]. In this regard, a variety of biomaterials can be used to generate tailored delivery systems that regulate inflammation and promote tissue regeneration.

## 6. Conclusions

Metabolic diseases are associated with increased morbidity and mortality from cardiovascular disease, which is the leading cause of death worldwide. Novel biomarkers for early diagnosis and prognosis and therapeutic strategies that prevent end-organ damage are urgently needed. EVs and exosomes, specifically, have been described as mediators in the pathogenesis of this cluster of disorders.

Increased levels of circulating exosomes observed in obesity, insulin resistance and diabetes reflect the importance of these EVs for signal transduction in metabolic regulation. Specific proteins and RNAs enclosed in exosomes relate to critical signaling pathways in glucose and lipid metabolism. Furthermore, cardiac function can also be regulated by exosomal cargo. Changes in blood pressure and vascular damage due to glucotoxicity and lipotoxicity also induce the release of exosomes with specific cargoes that has been shown to ameliorate or further propagate tissue damage.

Exosomes have been associated with the inflammatory response in the setting of cardiometabolic disease. As is already known, inflammation links metabolic and cardiovascular dysfunction. Adipocytes, endothelial cells, and cardiomyocytes release exosomes that can modulate the differentiation and function of macrophages and lymphocytes, either to restore tissue homeostasis or to further increase inflammation. Furthermore, exosomes can transfer components and modulators of inflammatory pathways across the immune system.

Because of their functional role and specific content, exosomes have been proposed as a non-invasive biomarker for diagnosis and prognosis. Evidence shows that these EVs have the potential to be used in determining the risk of developing metabolic abnormalities. Furthermore, these EVs can also be proposed for the early diagnosis of cardiometabolic disorders and to assess disease progression. Exosome assessment may be a companion diagnostic of classical markers of metabolic disease, such as blood glucose levels, blood lipid levels and blood pressure.

As it has been mentioned, exosomes are also nanocarriers that can be loaded with therapeutic compounds for specific delivery to target tissues. Either natural or synthetic, these EVs can be modified to generate highly tailored nanocarriers that can deliver their cargo to specific cell types. Recent progress in biomaterials has also shown that exosomes are stable in these systems, preserving their functional properties, which includes tissue regeneration.

For their part, stem-cell derived exosomes may also increase the efficacy of regenerative medicine strategies, either by improving cell therapy or as a cell-free alternative. In this work we have summarized valuable evidence of the functional role of exosomes in cardiometabolic disease, although we are only beginning to understand the regulatory capacity of these EVs in cell function and their therapeutic potential.

## Data Availability

Not applicable.
